# Revisiting the Institut Mutualiste Montsouris Difficulty Classification of Laparoscopic Liver Resection with the Data from a Personal Series—Evaluations for the Difficulty of Left Medial Sectionectomy and Length of Hospital Stay

**DOI:** 10.3390/jpm14030232

**Published:** 2024-02-22

**Authors:** Zenichi Morise

**Affiliations:** Department of Surgery, School of Medicine, Fujita Health University, Okazaki Medical Center, 1 Gotanda Harisakicho, Okazaki 444-0827, Japan; zmorise@fujita-hu.ac.jp; Tel.: +81-564-64-8800; Fax: +81-564-64-8135

**Keywords:** laparoscopic liver resection, difficulty score, left medial sectionectomy, length of stay, anatomical liver resection, segmentectomy, sectionectomy, hemihepatectomy

## Abstract

The IMM (Institut Mutualiste Montsouris) difficulty classification for laparoscopic liver resection is based only on the type of surgical procedure. It is useful for assessing outcomes and setting benchmarks in a group sharing the same indications. There is, however, no left medial sectionectomy in the system. Its difficulty was evaluated using the same methodology as IMM with the data from a personal series. Furthermore, length of hospital stay was evaluated as the representative of short-term outcomes. IMM scores of our right and left hemihepatectomies, right anterior sectionectomy, and segment 7 or 8 segmentectomies are 3. That of left medial sectionectomies is 2, the same as right posterior sectionectomy and segment or less anatomical resections. Those of left lateral sectionectomy and partial resection are 0. The group with a score of 3 was divided into two groups—with and without extended hospital stays (extended only for right hemihepatectomies and right anterior sectionectomies). The difficulty of medial sectionectomy was positioned at the same level as posterior sectionectomy and segment or less anatomical resections. The result from the second evaluation may indicate that there are other factors with an impact on difficulty related to short-term outcomes, other than intraoperative surgical difficulty from the procedure itself.

## 1. Introduction

Laparoscopic liver resection (LLR) was first introduced in the early 1990s [1,2,3], and expanded rapidly with instrumental and technical improvements [4], with two international consensus conferences [5,6] and four world congresses of the International Laparoscopic Liver Society [7]. It is now a common surgical procedure performed in many hospitals; however, to ensure a safe dissemination of the procedure, an assessment of the difficulty of a planned LLR and a clearly defined training path for each specific procedure are needed. The difficulty of each LLR, which is complicated due to the involvement of numerous factors and is subjective in some aspects, can be hard to define. The first difficulty score-setting study, conducted by Ban et al. [8], defined the difficulty of LLR using multiple variables, including: tumor location; tumor size; proximity to the major vessels; liver function; and type of LLR. The difficulty was scored based on multiple factors in addition to the surgical procedure (type of LLR), because such factors could influence the surgical difficulty, even when the same type of LLR is performed. This original three-level difficulty scoring system had problems, however, such as lacking a segment 1 and having no separation between segments 4a and b in the tumor location category, as well as lacking a category for hand-assisted and hybrid methods. It was refined, therefore, to the IWATE criteria, which included four classification levels, during the Second International Consensus Conference on LLR [9,10]. The major strength of the revised scoring system was that it could be used among different institutions with different indications of LLR for liver tumors and chronic liver conditions. However, it still remains difficult to integrate all of the potential risk factors in order to set an objective prediction of technical difficulty. Since there are more detailed factors and complex scenarios affecting the outcome of each individual case [11,12,13], several other scoring systems have been developed [14,15]. Kawaguchi et al. [16] presented a simple difficulty classification (Institut Mutualiste Montsouris [IMM] classification) system, based only on the type of surgical procedure, which they established based on the following data of a single institute: operation time; blood loss; and conversion rate to laparotomy. When this classification system is used for cases with the same indication for a given type of LLR, certain other factors, such as tumor size and location, liver function, and proximity to the major vessels, do not have a large effect on determining the difficulty of each procedure, as they have been already considered when selecting the type of liver resection. Therefore, although the IWATE criteria are more precise, and useful for the universal assessment of the planned LLR’s difficulty and selecting a primary surgeon, the IMM classification is useful at the time of assessing the surgical outcomes and setting benchmarks for a certain type of LLR in a single institute or a group of institutes sharing the same indications.

## 2. Left Medial Sectionectomy in IMM Classification System

According to the Brisbane 2000 Nomenclature [17], the left medial section of the liver consists of only one segment (segment 4), while the other sections (left lateral, right anterior, and right posterior) all consist of two segments. The cranial (S4a) and caudal (S4b) parts of segment 4, however, are often handled separately, especially in LLR. In the IMM classification system [16] which classifies the difficulty of each LLR procedure based only on the resection type, wedge (partial) resections of S4b belong to anterolateral segment resections, and S4a to posterosuperior segment resections. There is, however, no left medial sectionectomy (LMS; S4 segmentectomy) in the IMM classification system. Left lateral and right posterior sectionectomies are both defined, and a central hepatectomy is defined as the resection of segments 5 and 8 (right anterior sectionectomy) or segments 4, 5, and 8 (resection of two sections, right anterior plus left medial sections); however, there is no definition for the resection of segment 4 (medial section) alone. Although wedge (partial) resections of S4b and S4a are included in anterolateral segment and posterosuperior segment resections, respectively, the S4 segmentectomy (LMS) is not clearly defined, and can theoretically be classified as both the posterosuperior (Group III) and anterolateral (Group II) segmentectomies.

Tumors in S4b tend to be treated with partial or small anatomical resections. Although small tumors in S4a can be resected by partial resection, large tumors or tumors invading/contacting a major hepatic vein in S4a should be treated with a left lobectomy with a concurrent resection of the left hepatic vein (LHV) or a central bi(right anterior plus left medial)-sectionectomy with a concurrent resection of the middle hepatic vein (MHV). LMS of the liver is, however, still performed on occasion, such as for hepatocellular carcinoma (HCC) in the deep part of the section with limited residual liver function. The present manuscript includes six cases of LMS, which were used in an effort to try to evaluate the difficulty of the procedure using the same methodology as used in the paper in which the IMM classification system was presented [16].

## 3. Methods Used for Determining IMM Classification and in the Present Study

The IMM classification for the difficulty of a given LLR is determined by adding the points from the median values of the operation time, blood loss, and conversion rate for each type of LLR. One point is allocated for each of the following, as applicable: operative time of ≥190 min (median of all cases); blood loss ≥ 100 mL; or a conversion rate ≥ 4.2%. The aforementioned values are the median of all cases, and each LLR procedure received a score of 0–3 points, after which all of the LLRs were divided into three groups. The study presenting the IMM classification system stated that “the operative time, total intraoperative blood loss, and conversion rate were evaluated to address surgical difficulty with a certain degree of objectivity, because surgical difficulty can be reflected in a combination of these intraoperative factors” [16].

This study conformed to the ethical guidelines of Declaration of Helsinki and was retrospective in nature. Approval from the ethics committee of Fujita Health University was obtained (HM17-164, approved on 2 September 2017).

1. *Evaluation of the difficulty of each type of LLR, including LMS, using the data from a personal case series and the IMM classification system*

Using the same methodology as the IMM classification system, the difficulty of each type of LLR, including LMS, was evaluated in the present study using a personal (ZM operated) case series, in which the same indications and surgical techniques were applied. Since IMM classification is very useful at assessing the outcomes of surgeries performed under the same conditions, the data of the LLRs performed by a single surgeon for the same indications were analyzed in the present study.

Since there were a relatively large number of patients in the aforementioned series who underwent segment or less anatomical resections (such as segment 8a, 8c, 4a, or 4b resections) for HCC with chronic liver disease (CLD), the group of segment or less anatomical resections, excluding segment 7 or 8 segmentectomy (anterolateral segmentectomy or smaller anatomical resection), was defined and evaluated in the present study. Furthermore, there were no segment 1 segmentectomies in the aforementioned series. Posterosuperior segmentectomies in IMM classification were re-labeled as segment 7 or 8 segmentectomies. Extended hemihepatectomies were included in hemihepatectomies, and extended sectionectomies, which are not defined in the IMM classification system, were included in the sectionectomies in the present study. Finally, the LLR patients were divided into the following groups: right hemihepatectomy; left hemihepatectomy; right posterior sectionectomy; right anterior sectionectomy; left medial sectionectomy; left lateral sectionectomy; segment 7 or 8 segmentectomy; segment or less anatomical resection, excluding segment 7 or 8 segmentectomy; and partial resection.

After April 2010, when our technique of LLR, especially for liver parenchymal transection, was well established, 231 LLRs were performed by a single surgeon (ZM), 68 between April 2010 and December 2014, 76 between January 2015 and December 2019, and 87 between January 2020 and August 2023. Of the 231 patients, 50% had CLDs, 14% and 24% had portal hypertension and histologically proven liver cirrhosis, respectively, and 22% had a history of previous liver resection. Among the included procedures, there were 12 right hemihepatectomies, 17 left hemihepatectomies, 19 right posterior sectionectomies, eight right anterior sectionectomies, six left medial sectionectomies, 14 left lateral sectionectomies, nine segment 7 or 8 segmentectomies, 36 segment or less anatomical resections, excluding segment 7 or 8 segmentectomy, and 119 partial resections.

2. *Evaluation of the difficulty using the IMM classification system plus the length of the postoperative hospital stay*

In the second evaluation of the present study, the length of the postoperative hospital stay was evaluated as the representative value of short-term postoperative outcomes. Patients can be hospitalized for postoperative rehabilitation for 10–14 days, based on the coverage afforded by the Japanese social insurance system. Since the patients in the aforementioned series were mostly of older age groups (median age, 70 years; 13% > 80 years old) and often with a history of CLD (50% of all cases) and previous liver resections (22%), most of the patients were put on the perioperative cancer rehabilitation program list. The aim of the rehab program was that patients achieved almost normal performance status (without analgesics) and normal oral intake through discharge. Finally, the median length of stay of 12 days for patients who underwent a partial resection or left lateral sectionectomy (LLS) was used as the basic length of stay post-LLR in the present study (although the length was sometimes longer, even among Japanese hospitalizations). Since an extended length of stay is usually due to short-term morbidity, the extended length can be the representative value of postoperative short-term outcomes.

In the present study, we aimed to classify LLR procedures (including LMS) with the same methodology as the IMM classification system, while adding the length of postoperative hospital stay as an evaluated factor of a postoperative outcome indicators in further evaluations.

## 4. Results

### Backgrounds Factors in Each Group

Table 1 shows the backgrounds of patients in each group. All values—tumor number, tumor size, and indocyanine green 15-min retention (ICGR15)—are the medians from the group.

The median values of tumor number in right hemihepatectomies and segment 7 or 8 segmentectomies (2.5 and 2.0, respectively) were above the median value of all cases (1.0). Those of tumor size in right and left hemihepatectomies were 60.5 and 80.0 mm, respectively, and that in anterior sectionectomies was 50.0 mm. Only median values of tumor size in segment or less anatomical resections, excluding segment 7 or 8 segmentectomy and partial resections (20.0 and 23.0 mm, respectively) were below that for all cases (25.0 mm). Only median values of ICGR15 in segment or less anatomical resections, excluding segment 7 or 8 segmentectomy and partial resections (13.9 and 14.8%, respectively), were above those for all cases (11.7%).

In general, right hemihepatectomies were performed for patients with good liver function (33% of the cases, <50% of all cases, had background CLDs) and large (median tumor size of 60.5 mm, larger than median size 25.0 mm for all cases) and multiple tumors. Left hemihepatectomies were performed for patients with good liver function (41% of cases had CLDs) and large (80 mm, median value of tumor sizes) tumors. Right anterior sectionectomies were performed for patients with poor liver function (75% with CLDs) and large (50 mm, median value of tumor sizes) tumors. Right posterior, left medial, and left lateral sectionectomies were performed for patients with relatively large (29.0, 35.0, and 38.5 mm, respectively, median value of tumor sizes) tumors and relatively good liver function (42, 50, and 36% with CLDs, respectively). Segment or less anatomical resections, excluding segment 7 or 8 segmentectomy, and partial resections were performed in patients with poorer liver function (59 and 52% with CLDs, respectively) and small (20.0 and 23.0 mm, respectively, median value of tumor sizes) tumors. The patients who underwent segment 7 or 8 segmentectomies had tumors larger in number (two) and moderate in size (26.0 mm, median value of tumor sizes), and poor liver function (56% with CLDs).

Furthermore, segment or smaller resections (segment 7 or 8 segmentectomy, segment or less anatomical resections, excluding segment 7 or 8 segmentectomy, and partial resections) were more frequently performed as a repeat liver resection (33, 33, and 27%, respectively were repeats, >22% of all cases). Of the included cases, 24% had histologically-proven liver cirrhosis, and 14% of all cases had portal hypertension (6/27, 3/14, and 22/119 cases in segment or less anatomical resections excluding segment 7 or 8 segmentectomy, left lateral sectionectomy, and partial resections, respectively, had portal hypertension).

1. *Difficulty score for each type of LLR, including LMS, based on the IMM classification system using data from personal case series*

Table 2 shows operation time, blood loss, conversion rate, and length of postoperative hospital stay in each group.

The median values of operation times for right and left hemihepatectomies, right posterior and anterior, and left medial sectionectomies, segment 7 or 8 segmentectomies, and segment or less anatomical resections, excluding segment 7 or 8 segmentectomy (498.0, 336.0, 444.0, 475.5, 492.0, 424.0, and 351.0 min, respectively) are >the median of all cases (323.0 min). The median values of blood loss for right and left hemihepatectomies, right posterior and anterior, and left medial sectionectomies, segment 7 or 8 segmentectomies, and segment or less anatomical resections, excluding segment 7 or 8 segmentectomy (603.0, 205.0, 310.0, 582.5, 292.0, 160.0, and 150.0 mL, respectively) are >the median of all cases (108.0 mL). The conversion rates for right and left hemihepatectomies, right anterior sectionectomies, and segment 7 or 8 segmentectomies (8.3, 5.9, 12.5, and 11.1%, respectively) are > the median of all cases (3.0%). The median values for operation time, blood loss, and conversion rate for LLS and partial resections were all below the median of all cases.

According to the IMM classification system, the scores for right and left hemihepatectomies, right anterior sectionectomies, and segment 7 or 8 segmentectomies are all 3. The scores for left medial sectionectomies, right posterior sectionectomies, and segment or less anatomical resections, excluding segment 7 or 8 segmentectomy, were all 2. Those of left lateral sectionectomies and partial resection were 0. In the IMM classification system, resection types with score 3 were categorized as IMM grade III, advanced level. Those with scores of 2 and 0 were categorized as grades II and I, intermediate level and beginning least complex level, respectively (Table 3).

2. *Classification of difficulty using the IMM system plus length of postoperative hospital stay*

The median values for length of postoperative hospital stay for right hemihepatectomies, right posterior and anterior, and left medial sectionectomies, and segment or less anatomical resections, excluding segment 7 or 8 segmentectomy (28.0, 15.0, 22.5, 28.5, and 16.0 days, respectively) were > the median value of all cases (14.0 days).

When these values were evaluated using the same style of evaluation as the IMM classification system for operation time, blood loss, and conversion rate, the group with a score of 3 (grade III) was divided into two groups—with and without extended hospital stays (extended for right hemihepatectomies and right anterior sectionectomies, although not for left hemihepatectomies and segment 7 or 8 segmentectomies). The group with a score of 2 (grade II, left medial sectionectomies, right posterior sectionectomies, and segment or less anatomical resections, excluding segment 7 or 8 segmentectomy) had extended hospital stays, while that with a score of 0 (grade I, left lateral sectionectomies and partial resections) did not.

3. *Backgrounds of the patients who underwent right hemihepatectomy*

The median value of LOS of the right hemihepatectomy group in the present study (12 cases) was 28 days, which is even longer than the Japanese insurance system covering postoperative rehabilitation.

Although five of 12 cases were discharged in 2 weeks, there were four patients with an LOS around 4 weeks and three patients with an LOS > 4 weeks. Since the cases were selected for the operation of the most senior surgeon (ZM), all the patients had complex backgrounds. In these seven patients with extended stays, two patients had a damaged liver from previous intensive chemotherapies of 12 cycles of FOLFOX and repeated transarterial chemoembolization for hepatocellular carcinoma during intravenous repeated chemotherapy for lung cancer, respectively. There was a patient who had a conversion to open procedure due to a 20 cm hepatocellular carcinoma packed into the subphrenic space and who developed postoperative pleural effusion. Another patient developed postoperative ileus after a simultaneously performed right hemicolectomy. There was another patient with tumor thrombus in the main trunk of the portal vein preoperatively and also three aged patients in their 80s were included.

## 5. Discussion

The present study was conducted based on the IMM classification system of LLR difficulty using the data from a personal series (surgeon, ZM) for which the same indication and surgical technique were applied. We evaluated three groups (scores 0, 2, and 3) of procedural difficulties, determined based on the IMM classification system (Table 3), in which LLR types were divided as follows: Grade I (0 points) included partial resections and left lateral sectionectomies; Grade II (2 points), anterolateral segmentectomies and left hemihepatectomies; and Grade III (3 points), posterosuperior segmentectomies, right posterior sectionectomies, right hemihepatectomies, central hepatectomies (including right anterior sectionectomies), and extended left/right hemihepatectomies. In the present study, grade I was the same as IMM grade I; however, left hemihepatectomies were classified as grade III, instead of grade II, as in the IMM system. Additionally, right posterior sectionectomies were classified as grade II, instead of grade III. In the present study, left hemihepatectomies had large tumors (median value of tumor size, 80.0 mm) and included extended left hemihepatectomies. The cases of left hemihepatectomies (grade II in IMM) and extended left hemihepatectomies (grade III in IMM) were merged into the left hemihepatectomy group in the present study, due to their small numbers. Hence, left hemihepatectomies are categorized as grade III in the present study. Right posterior sectionectomies were one of the earliest established procedures in our experience [18], during which patients are put in a left lateral position, and the liver parenchymal transection is performed prior to mobilization of the posterior section from the retroperitoneum. In this procedure, the resected posterior section is fixed to the retroperitoneum and the residual liver sinks down to the left due to gravity. Therefore, a well-opened stable transection surface is acquired and the most difficult part, handling the horizontal transection plane beneath the heavy and large right liver in the supine position, is cleared. This may be why right posterior sectionectomies are categorized as grade II in the present study.

LMS is also categorized as grade II in the present study, the same difficulty as right posterior sectionectomies (in our series) and segment or less anatomical resections, excluding segment 7 or 8 segmentectomy (anterolateral segmentectomy or smaller anatomical resection). Right and left hemihepatectomies, right anterior sectionectomies, and segment 7 or 8 segmentectomy are considered more difficult. The difficulty of each LLR procedure is defined by specific factors, such as size of transected area, need for handling and processing major vessels, difficulty in acquiring a well-opened transection plane with traction, mobilization and removal of the resected liver, and difficulty approaching the working space. In LMS, the transection area is large. MHV dissection is required on the right side-transection surface, and the anterior Glissonian pedicle requires processing. However, the transection planes on the right and left sides are both vertical, and the difficulty of acquiring a well-opened transection plane with traction is low. Furthermore, mobilization and removal of the resected liver is not needed, and the difficulty approaching the working space is also low. In other words, the approach to LMS is similar to anterolateral segmentectomies or partial resections, with low difficulty, but a large transection area with major vessels dissection is needed. Therefore, LMS difficulty was evaluated as grade II, which is thought to be reasonable. However, there were only six patients who underwent LMS in the present study. Further investigation with large number of cases should be needed.

In the present study, the evaluation of another variable of length of hospital stay (LOS), which can reflect postoperative short-term outcomes, was performed. LOS was evaluated using the same IMM style as the other factors. Each type of LLR was evaluated to determine whether its median value of LOS was longer than that of all cases. The grade III group was divided into two groups—with and without extended hospital stays (right hemihepatectomies and right anterior sectionectomies were extended, but not left hemihepatectomies and segment 7 or 8 segmentectomies) for the present study evaluating a personal (surgeon, ZM) case series in which same indications and surgical techniques were applied. This may indicate that there are other factors with an impact on difficulty related to short-term outcomes, other than intraoperative surgical difficulty (complexity) from the procedure itself. For universal usage for preoperative judgement of matching a case to a surgeon based on level of training, a more complex scoring system, such as the IWATE criteria, including liver function and proximity to major vessels [9,19], and Southampton difficulty score, including neoadjuvant chemotherapy and previous open liver resection [14], could be suitable, even though the IMM classification system is simple and useful for assessing the surgical outcomes and setting benchmarks for a certain institute or a group of institutes sharing the same indications.

**Figure 1:**
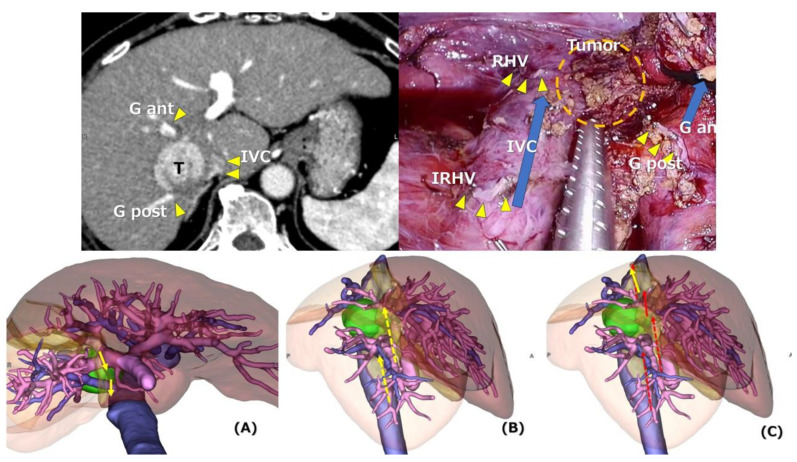
Preoperative simulation and intraoperative navigation using preoperative imaging. **Upper left**: A 73-year-old woman with non-alcoholic steatohepatitis-based liver cirrhosis developed a 3.5 cm hepatocellular carcinoma in segments 7-1r-8c. Since her liver function was not sufficient for right hemihepatectomy, she underwent LLR of posterior section + segment 1r + dorsal part of segment 8. **Upper right**: Image of postoperative findings shows the stump of right hepatic vein (RHV), inferior right hepatic vein (IRHV), and posterior Glissonian pedicle (G post) (all are shown by yellow arrow heads). IVC surface (IVC) and dorsal surface of the anterior Glissonian pedicle (G ant) are dissected and exposed (blue arrows). The tumor, which existed in the area shown by the orange dotted circle (Tumor), was resected by planned LLR of posterior section + segment 1r + dorsal part of segment 8. Operation time was 340 min and blood loss was 425 mL. She discharged at nine postoperative days without any morbidity. Preoperative simulation was performed using the reconstruction images from preoperative computed tomography. The green ball is the tumor, blue vessels are hepatic veins and IVC, and purple vessels are portal veins. (**A**) is a caudal view of the image. She had a thick inferior right hepatic vein. In the first step of the procedure, the posterior Glissonian pedicle and inferior right hepatic vein were divided. (**B**,**C**) are lateral views of the image. In the second step of the procedure (**B**) during the liver parenchymal transection, the dorsal surface of anterior Glissonian pedicle was dissected dividing the branched tissue to the dorsal direction, and dissection of the surface of the IVC was performed. In the third step of the procedure (**C**) after the completion of liver parenchymal transection, the right hepatic vein was divided and, thereafter, resected liver was dissected from retroperitoneum and extracted.

Further studies are needed for adding LOS to the difficulty classification. For example, the median value of LOS of the right hemihepatectomy group in the present study (12 cases) was 28 days, which is even longer than the Japanese insurance system covering postoperative rehabilitation. The LOS of right hemihepatectomy group was much longer especially with the conditions described above in case report section. However, recent consecutive patients were all discharged in two weeks. Although the indications and also surgical techniques were not changing much, there had been several points of developments in the other part of perioperative settings, such as preoperative simulation and intraoperative navigation using preoperative imaging [Figure 1] [20,21]. There are also other several developments around LLR during recent years, such as landmark (hepatic veins, etc.)-guided small anatomical resection [22], indocyanine green (ICG) fluorescence-guided anatomical resection/tumor identification [23,24,25], and LLR with a Glissonian approach to more peripheral smaller branches from the hilum (cone unit resection) [26,27]. Robot-assisted LLR is also an important emerging tool under discussion [28,29]. It could be advantageous for complicated cases, such as cases exposing a wide range of Glissonian pedicles and major hepatic veins and with need for vessel reconstruction. There are also emerging minimally invasive treatments besides LLR, like radiation segmentectomy with radioembolization [30]. In the near future, the difficulty of each case for LLR should be evaluated and compared with the others more strictly in its risks and benefits, and also considered in regard to young surgeons’ training path and the safe dissemination of the procedure. The present study was conducted to evaluate LMS difficulty and the impact of LOS on the IMM difficulty scoring-system, which could be additional information for such judgements. However, there were only six patients who underwent LMS, and the data of LOS were retrieved from the patients under this study’s specific conditions mentioned before. Further investigation of difficulty scoring systems for precise selection of the patients, for each surgeon, and each LLR with a large number of cases under universal conditions is needed.

## Figures and Tables

**Table 1:** Background Factors in Each Group by Resection Type. Values of T number, T size, and ICGR15 are median values of the group.

LLR Type (n)	T Number	T Size (mm)	ICGR15	CLD (%)	Repeat Case (%)
Right Hep (12)	2.5	60.5	8.5	33	0
Right Anterior (8)	1.0	50.0	10.3	75	0
Left Hep (17)	1.0	80.0	10.4	41	16
Seg 7 or 8 (9)	2.0	26.0	10.8	56	33
Right Posterior (19)	1.0	29.0	8.7	42	5
Left Medial (6)	1.0	35.0	11.0	50	17
Seg or Less Anatomical excluding Seg 7/8 (27)	1.0	20.0	13.9	59	33
Left Lateral (14)	1.0	38.5	10.0	36	21
Partial Resection (119)	1.0	23.0	14.8	52	27
ALL (231)	1.0	25.0	11.7	50	22

LLR: laparoscopic liver resection. T: tumor; ICG-R15: 15 min value of the retention rate (%) during indocyanine green loading test; CLD: rate of the cases with chronic liver diseases among all; Repeat Case: rate of the cases with previous liver resection. Right Hep: right hemihepatectomy; Right Anterior: right anterior sectionectomy; Left Hep: left hemihepatectomy; Seg 7 or 8: segment 7 or 8 segmentectomy; Right Posterior: right posterior sectionectomy; Left Medial: left medial sectionectomy; Seg or Less Anatomical excluding Seg 7/8: segment or less anatomical resection excluding segment 7 or 8 segmentectomy; Left Lateral: left lateral sectionectomy; ALL: data from all patients.

**Table 2:** Postoperative Outcomes in Each Group by Resection Type. Values of Operative Time, Blood Loss, and Length of Stay are median values of the group. Values in [ ] show median values from the paper of IMM classification [16]. In the “Partial Resection” row, left values inside [ , ] show median values of partial resection in anterolateral segments, and right values show those in posterosuperior segments from the paper of IMM classification [16].

LLR Type (n)	Operative Time, min	Blood Loss, mL	Conversion Rate, %	Length of Stay, Day
Right Hep (12)	498.0 [240]	603.0 [215]	8.3 [5.1]	28.00
Right Anterior (8)	475.5 [205]	582.5 [300]	12.5 [18.2]	22.50
Left Hep (17)	336.0 [210]	205.0 [120]	5.9 [3.8]	14.00
Seg 7 or 8 (9)	424.0 [198]	160.0 [110]	11.1 [6.5]	12.00
Right Posterior (19)	444.0 [300]	310.0 [350]	0.0 [14.3]	15.00
Left Medial (6)	492.0	292.0	0.0	28.50
Seg or Less Anatomical excluding Seg 7/8 (27)	351.0	150.0	0.0	16.00
Left Lateral (14)	261.5 [135]	50.0 [15]	0.0 [0]	12.00
Partial Resection (119)	246.0 [120, 172]	50.0 [0, 50]	1.7 [0, 1.7]	12.00
ALL case (231)	323.0 [190]	108.0 [100]	3.0 [4.2]	14.00

LLR: laparoscopic liver resection. Length of Stay: length of postoperative hospital stay. Right Hep: right hemihepatectomy; Right Anterior: right anterior sectionectomy; Left Hep: left hemihepatectomy; Seg 7 or 8: segment 7 or 8 segmentectomy; Right Posterior: right posterior sectionectomy; Left Medial: left medial sectionectomy; Seg or Less Anatomical excluding Seg 7/8: segment or less anatomical resection excluding segment 7 or 8 segmentectomy; Left Lateral: left lateral sectionectomy; ALL: data from all patients.

**Table 3:** IMM style Difficulty Scores, Grade, and Level of Expertise in Each Group of Resection Type.

LLR Type (n)	Original IMM Score	IMM Score in Present Study	Original IMM Grade	IMM Grade in Present Study	The Levels of Expertise
Right Hep (12)	3	3	III	III	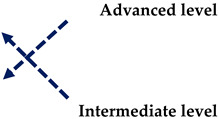
Right Anterior (8)	3	3	III	III	
Left Hep (17)	***2***	***3***	***II***	***III***	
Seg 7 or 8 (9)	3	3	III	III	
Right Posterior (19)	***3***	***2***	***III***	***II***	
Left Medial (6)		2		**II**	
Seg or Less Anatomical excluding Seg 7/8 (27)	2	2	II	II	
Left Lateral (14)	0	0	I	I	**Beginning and least complex level**
Partial Resection (119)	0	0	I	I	
ALL case (231)					

LLR: laparoscopic liver resection. IMM score: score from IMM classification system; IMM grade: score from IMM classification system; Original: score and grade from IMM original paper [16]. Right Hep: right hemihepatectomy; Right Anterior: right anterior sectionectomy; Left Hep: left hemihepatectomy; Seg 7 or 8: segment 7 or 8 segmentectomy; Right Posterior: right posterior sectionectomy; Left Medial: left medial sectionectomy; Seg or Less Anatomical excluding Seg 7/8: segment or less anatomical resection excluding segment 7 or 8 segmentectomy; Left Lateral: left lateral sectionectomy; ALL: data from all patients.

## Data Availability

Dataset available on request from the authors.
